# The role of digital health interventions for adults with tuberculosis: a network meta-analysis of randomized controlled trials

**DOI:** 10.3389/fmed.2026.1840258

**Published:** 2026-06-09

**Authors:** Yuanjie Duan, Juan Liu, Shiyu Lin, Xi Li, Dan Luo, Lemei Zhu

**Affiliations:** 1 Hunan Key Laboratory of the Research and Development of Novel Pharmaceutical Preparations, Changsha Medical University, Changsha, China; 2 Hunan Provincial University Key Laboratory of the Fundamental and Clinical Research on Neurodegenerative Diseases, Changsha Medical University, Changsha, China; 3 School of Public Health, Changsha Medical University, Changsha, China

**Keywords:** adherence, digital health intervention, meta-analysis, treatment success, tuberculosis

## Abstract

**Background:**

Incomplete adherence to tuberculosis treatment increases the risk of delayed sputum culture conversion in the community, as well as elevated risks of treatment failure, recurrence, and the development or amplification of drug resistance. Through network meta-analysis, we aimed to comprehensively analyze the effects of digital health interventions for patients with tuberculosis.

**Methods:**

PubMed, Cochrane Library, Embase and Web of Science for randomized controlled trails that examined the efficacy of digital health intervention for tuberculosis treatment up to 18 May 2026. The main outcome included treatment success and adherence. Stata (version 17) and R software (version 4.3.1) were used for the data analysis.

**Results:**

From 17,643 publications, we included 29 randomized controlled trials involving 17,800 participants for quantitative analysis. Overall, digital health interventions showed a statistically higher treatment success rate than directly observed therapy (RR 1.03; 95% CI 1.00–1.07). Then a network meta-analysis of each intervention was conducted. Regarding treatment success, apart from video observed therapy showing significantly better outcomes compared to directly observed therapy (RR 1.18; 95% CI 1.02–1.37) and double-way short message service (RR 0.80; 95% CI 0.67–0.95), there was no statistically significant difference observed between medication event reminder monitor systems, monitors, single-way short message services, double-way short message service, video observed therapy, phone calls, and directly observed therapy. In terms of adherence, video observed therapy also demonstrated significantly higher adherence rates compared to other interventions. Additionally, video observed therapy was ranked as the top digital health intervention for achieving both treatment success and adherence.

**Conclusion:**

Video observed therapy has excellent effects on treatment success and adherence than other digital health interventions. Given its excellent efficacy, video observed therapy should be given more prominence in clinical care, especially for individuals with low adherence, where resources allow.

## Introduction

According to the World Health Organization Global Tuberculosis Report 2025, an estimated 10.7 million people fell ill with tuberculosis globally in 2024, and an estimated 1.23 million people died from the disease (1) 2). TB remains one of the top 10 causes of death worldwide and the leading cause of death from a single infectious agent — above many other infectious diseases including COVID-19 in recent years (2). The treatment of tuberculosis mainly relies on standardized multi-drug therapy, including a 2-month intensive phase followed by a 4-month maintenance phase (3). Factors such as long-term medication, adverse reactions, and mistaken belief in cure after symptom relief may lead to decreased patient adherence (4). Reduced adherence during tuberculosis treatment makes it difficult for patients to achieve optimal therapeutic outcomes, potentially resulting in persistent symptoms or relapse.

During the process of anti-tuberculosis treatment, directly observed therapy (DOT), which involves regular supervision of medication intake by healthcare workers or community workers, plays an important role in assisting patients to complete the entire treatment process (5–7). However, DOT has drawbacks including the need for significant human and financial resources, impacts on patient privacy and autonomy, limited accessibility and affordability, disputed effectiveness, and difficulty in covering all tuberculosis patients (8–10). With the advancement of technology, the emergence of digital health interventions has been proven to enhance the treatment outcomes of many chronic diseases, such as hypertension and type 2 diabetes (11–14). In the treatment of tuberculosis, numerous studies have also explored the effects of digital health interventions such as short message service (SMS), medication event reminder monitor system (MERM), video observed therapy (VOT) on improving tuberculosis treatment adherence (15). A 2018 meta-analysis reported that digital health intervention improved compliance and treatment success rates in tuberculosis treatment (16). However, this analysis was limited by the small number of studies included on digital health interventions (only 3 RCTs), high heterogeneity among studies (two on SMS messages, one on phone calls), and small sample sizes. Furthermore, due to the rapid advancement of technology, many studies have been published since that review, necessitating an update of the evidence. Importantly, despite the use of various digital health methods, such as SMS, smartphone applications, and monitors for interventions, there is currently no comprehensive comparison of their effectiveness and implementation, making it challenging to make informed decisions.

To assist clinicians in making decisions regarding which digital health interventions are more effective for self-management of tuberculosis, we conducted a network meta-analysis of randomized controlled trials to compare SMS, MERM, monitor, VOT, and phone call.

## Methods

This study was performed following the 2020 Preferred Reported Items for Systematic Reviews and Meta-analysis (PRISMA) guidelines. The study protocol was registered on PROSERPR.

### Search strategy and selection criteria

A comprehensive literature search was conducted on May 7, 2025 across several databases including PubMed, Cochrane Library, Embase, and Web of science with Mesh and broad search terms. The search was updated on 18 May, 2026. We also manually searched the reference lists of relevant review articles. After completing the initial research, we conducted the same search again to include the latest published studies. The detailed search strategy was in Supplementary Appendix.

The retrieved literature was imported into EndNote X9. After removing duplicate references, it was assessed for eligibility by two reviewers. According to the PICO principle, inclusion criteria were: patients receiving anti-tuberculosis treatment, intervention group using digital health intervention (such as SMS, Monitor, MREM, APP, etc.), and the control group not receiving corresponding digital health intervention, with reported treatment success or adherence. Exclusion criteria were: non-randomized controlled trials, and studies for which full text could not be retrieved.

### Data extraction

A comprehensive data extraction form was developed based on the guidelines outlined in the Cochrane Handbook for Systematic Reviews of Interventions. The form was piloted on a subset of the included studies before extracting the following data: study registration number; author details (names, affiliations, and conflicts of interest); study details (start and end date, country, design, purpose); participant characteristics (condition, severity of condition, comorbidities, inclusion and exclusion criteria, sample size, recruitment process, and demographics—age, sex, race, ethnicity, income, education, and remoteness of residence); intervention details (type, duration, frequency, and other details); primary and secondary outcomes; follow-up time; and conclusions. For each study, reach, adoption or uptake, and feasibility of interventions was also calculated (17–19).

### Outcomes

According to the WHO’s definitions of tuberculosis treatment outcomes, the indicators used were treatment success, adherence, and loss to follow-up. Treatment success is the sum of cure and treatment completion. Cure refers to a pulmonary TB patient with bacteriologically confirmed TB at the start of treatment, who is smear or culture negative in the last month of treatment and on at least one previous occasion. Treatment completion describes a TB patient who completed treatment without evidence of failure but lacks records of negative sputum smear or culture results, either due to tests not being done or results being unavailable. Adherence is defined by the standards set in the included studies, specifically as taking more than 80–90% of the prescribed doses. Loss to follow-up refers to a patient who did not start treatment or whose treatment was interrupted for two consecutive months or more.

### Quality assessment

Potential sources of bias in RCTs were assessed using Risk of Bias 2 (Rob2), a revised tool for assessing the risk of bias in randomized trials. Rob2 encompasses five key domains: 1. Randomization process; 2. Deviations from intended interventions; 3. Missing outcome data; 4. Measurement of the outcome; 5. Selection of the reported result. Within each domain, bias was evaluated and categorized as either low risk, some concerns, or high risk, depending on the circumstances and relevant evidence. Ultimately, the overall bias of each study was classified as either low risk, some concerns, or high risk, based on the comprehensive assessment of bias across the five domains. When there was a discrepancy in the assessment results for a certain domain, the outcome was resolved through discussion.

### Statistically analysis

In this study, data were analyzed using Stata (version 17) and R software (version 4.3.1). The study focused on binary outcomes, specifically calculating risk ratios (RR) with 95% confidence intervals (CIs) to measure effect sizes. Random effects model was employed to compare the effectiveness of various interventions.

Each intervention’s effectiveness was assessed using the surface under the cumulative ranking (SUCRA). Additionally, a ranking table was generated to compare the differences in effects between the intervention methods. Heterogeneity, indicating variability across trials, was defined with *I*^2^ > 50% indicating significant heterogeneity. The node-splitting method assessed local inconsistency within the network. Sensitivity analysis was conducted using both consistency and inconsistency models to assess result robustness. Funnel plots were used to evaluate publication bias.

Furthermore, conventional meta-analysis was performed for the primary outcome of treatment success, directly comparing digital health interventions and directly observed therapy. The quality of evidence was assessed by GRADE system (20). We performed a subgroup analysis of VOT by operational mode—synchronous (real-time, interactive) versus asynchronous (recorded, store-and-forward)—to assess their differential impact on outcomes.

### Role of the funding source

The funder of this study did not participate in its design, data collection, analysis, interpretation, or in the writing of the manuscript.

## Results

### Literature selection

**FIGURE 1:**
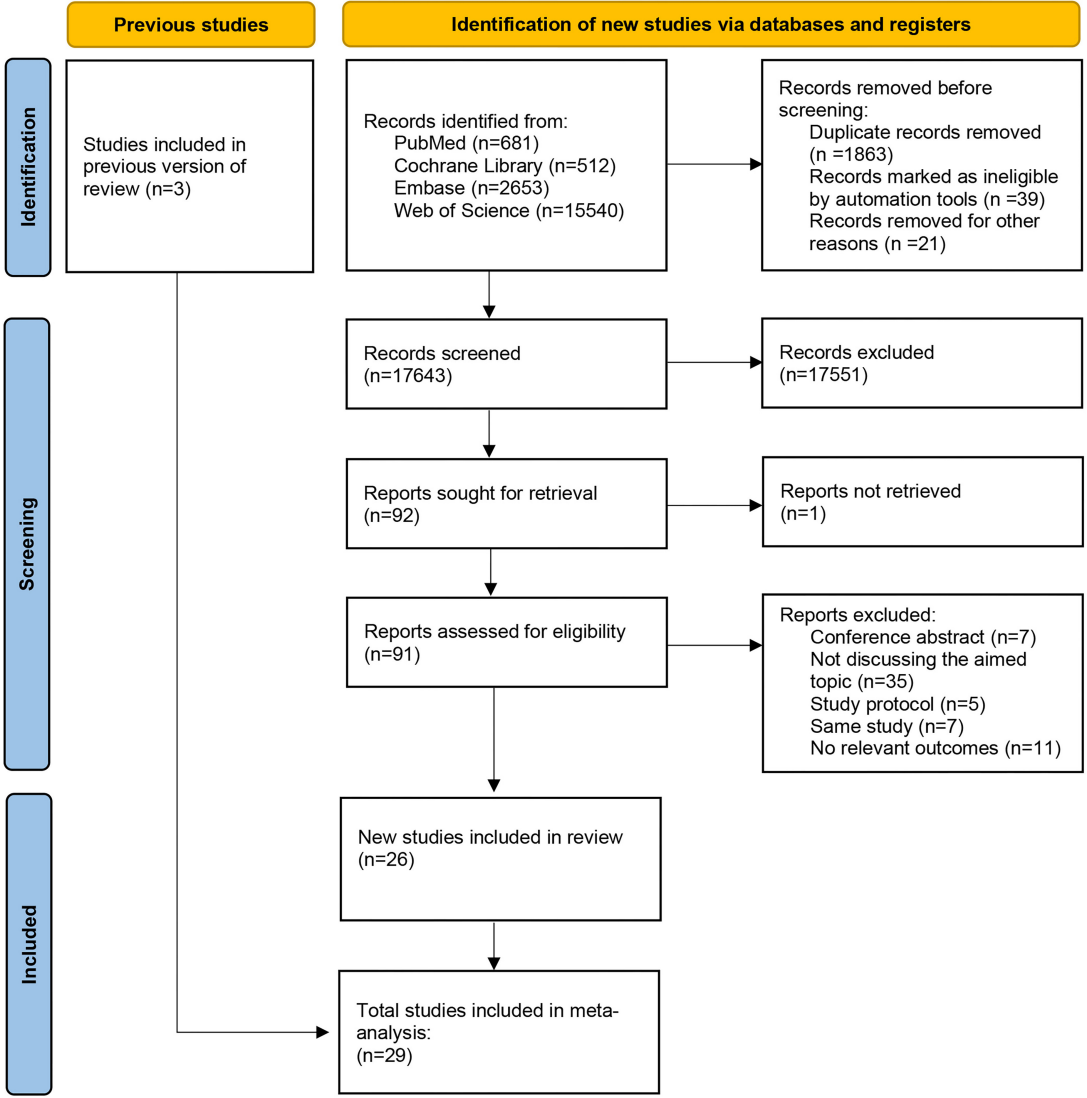
Flowchart of study selection.

Figure 1 illustrates the literature search and selection process. Initially, 17,643 literature records were identified from databases including PubMed (681), Cochrane Library (512), Embase (2,653), and Web of Science (15,540) for preliminary screening. Following the screening of titles and abstracts, 91 studies were selected for further evaluation via full-text review. Ultimately, 29 studies met the criteria for inclusion in the final analysis (Figure 1).

### Study characteristic

Table 1 provided a detailed overview of general information about the 29 included studies. These studies encompassed a total of 17,800 participants, with 8,839 receiving digital health interventions and 8,961 receiving Directly Observed Therapy (DOT). The digital health interventions included Medication Event Reminder Monitor system (MERM), monitoring, Short Message Service (SMS), Video Observed Therapy (VOT), and phone calls. SMS was categorized into single-way SMS and double-way SMS. Single-way SMS involved participants receiving SMS reminders without the need to reply to health workers, whereas double-way SMS required participants to both receive SMS reminders and respond to health workers each time. All studies compared digital health interventions with DOT. Of the 29 studies, 27 were two-arm trials, including 7 focusing on MERM, 1 on monitoring, 6 on single-way SMS, 5 on double-way SMS, and 2 on phone calls (21–32). There was one three-arm study focusing on single-way SMS and double-way SMS, and one four-arm study focusing on MERM, single-way SMS, and monitoring (33, 34). The reach of these interventions ranged from 3.2 to 88%, and their uptake ranged from 11 to 100%. Additionally, 22 studies reported high feasibility for the use of digital health interventions.

**TABLE 1:** Characteristics of included studies.

Study	Study design; duration; retention	Intervention	Treatment success	Conclusion	Percentage reach (randomly assigned proportion)	Uptake	Feasibility
MERM							
Acosta et al., 2022 (29)	RCT; 4 months; 102/106 (96%)	Electronic dispenser pillbox; Mobile phone network to monitor patient’s treatment; SMS message to reminder medication if the device was not opened at the hour scheduled for treatment	98% vs. 84.9%, *p* = 0.03	Effective	70.4 (50%)	NR	NR; reported high adherence
	RCT; 16 weeks; 61/61 (100%)	Wirelessly observed system allows date- and time-stamping of actual medication ingestion, SMS and phone call for missed medication	NR	NR	83.7% (67.2%)	100%	NR; reported on the satisfaction
Liu et al. (33)	Cluster RCT; 12 months; 4173/4231 (99%)	Daily SMS message only, medication only, combination of daily SMS reminders and monitor	MERM: 96.1% vs. 91.4%, p = 0.084; Monitor: 93.9% vs. 91.4%, *p* = 0.346; Single-way SMS: 91.2% vs. 91.4%, *p* = 0.991	Comparable	81.5% (73.4%)	41.5%	NR
Liu et al. (24)	Cluster RCT; 18 months; 2686/3074 (87%)	Medication event reminder, monitor patients’ medication	84% vs. 84%, *p* = 0.95	Comparable	20.1% (49.2%)	NA	NR; reported high adherence
Manyazewal et al. (26)	RCT; 2 months; 109/114 (96%)	medication event reminder and monitor device	NR	NR	33.8% (50%)	95.6%	NR; reported on adherence and effectiveness
Musiimenta et al. (23)	Pilot RCT; 6 months; 63/66 (95%)	Daily or weekly SMS to reminding patients taking medication, medication monitor	NR	NR	52.4% (68.2%)	100%	Feasible and acceptable and improve adherence
	Pilot RCT; 6 months; 80/100 (80%)	Monitor: send message or phone call if patients doesn’t open monitor at anticipated time	90% vs. 95.2%	Comparable	NR (50%)	60%	NR; reported high adherence, utility, and convenience
Wei et al., (22)	RCT; 6 months; 276/278 (99%)	Electronic medication monitor box, remind patients taking medicine	94% vs. 73%	Effective	41.9% (51.4%)	NR	NR; reported on high adherence and convenience
Monitor							
	RCT; 9 months; 166/189 (88%)	Medication monitors with or without feedback	87.8% vs. 88.2%, *p* = NA	NA	81% (31%)	NR	NR
VOT							
	RCT; 6 months; 396/405 (97%)	Take medicine under the administrator’s sight by smartphone application	96.1% vs. 94.6%, *p* = 0.474	Comparable	NR (50.1%)	NR	Feasible with greater reporting of adherence.
Iribarren et al. (28)	Pilot RCT; 6 months; 42/42 (100%)	Report daily self-administration of medication and complete urine test through app	95% vs. 81%, *p* = 0.14	Comparable	30.3% (48.6%)	57%	Feasible with greater reporting of adherence, convenience, satisfaction
Ravenscroft et al. (31)	RCT; 4 months; 175/197 (89%)	Patients send video about taking medication every day	94.1% vs. 90.3%, NA	NA	NR (49.7%)	11%	NR; reported high convenience and economy
	RCT; 2 months; 157/226 (69%)	Patients send video about taking medication every day	70% vs. 31%, *p* < 0.0001	Effective	41% (49.5%)	91%	Feasible based on function, acceptable, effective, cheaper
	RCT; 4 months; 123/130 (95%)	Patients send video about taking medication every day	73.5% vs. 69.4, *p* = NA	Comparable	6.7% (52.3%)	94.6%	Feasible based on economy
	Cluster RCT; 2 months; 114/128 (89%)	Patients send video about taking medication every day	73% vs. 62%, *p* = 0.17	Comparable			
Single-way SMS							
	RCT; 6 months; 178/279 (64%)	Daily SMS reminders which changed every 2 weeks	63.5% vs. 62%, *p* = 0.79	Comparable	NR (49.1%)	NR	NR; reported high satisfaction in general management and supported provided for adherence
	RCT; 6 months; 120/122 (98%)	Daily SMS reminders	NR	NR	NR (50.8%)	98%	Feasible with high adherence
	Cluster RCT; 6 months; 350/350 (100%)	One SMS message per day every morning to remind taking medication and reexamining periodically, TB-related knowledge	96.25% vs. 86.84%, *p* = 0.002	Effective	NR (45%)	NR	NR, reported on high adherence
Farooqi et al. (21)	RCT; 6 months; 148/148 (100%)	Daily SMS message to remainder in initial 2 months	94.6% vs. 93.2%, *p* = 0.983	Comparable	NR (50%)	NR	NR
Kibu et al. (34)	RCT; 6 months; 238/294 (81%)	Single-way SMS (reminding patients only), double-way SMS (patients need to reply to SMS message)	NR	NR	NR (66.7%)	NR	NR; Reported high adherence
	RCT; 2 months; 90/90 (77%)	SMS message to motivate patients’ drug consumption	NR	NR	NR (50%)	80%	NR; reported high adherence
Louwagie et al. (27)	RCT; 6 months; 497/574 (87%)	Three counseling sessions; SMS message giving information and augmenting motivation or behavior skills	67.8% vs. 70.1%, OR = 0.90 (95%CI 0.64 to 1.27)	Comparable	41.9% (49%)	NR	NR
MERMDouble-way SMS							
Cattamanchi et al. (30)	Cluster RCT; 8 months; 1453/1913 (76%)	Medication blister packs, daily SMS, confirm dosing by making daily phone calls to the system	72.7% vs. 70.9%, *p* = 0.87	Comparable	80% (40%)	NR	NR
Iribarren et al. (28)	Pilot RCT; 6 months; 26/37 (70%)	Send SMS after taking medication, twice weekly educational texts	94.4% vs. 89.5, *p* = NA	NA	30.3% (48.6%)	57%	Feasible with greater reporting of adherence, convenience, satisfaction
	RCT; 12 months; 336/358 (94%)	A weekly SMS message, patients need respond to SMS messages	79.4% vs. 81.4%, *p* = 0.608	Comparable	42.7% (47.5%)	62.8%	NR
	RCT; 6 months; 2197/2207 (99%)	SMS message: encourage patients to take medicine and ask for patients to respond	83% vs. 83%, *p* = 0.782	Comparable	57.6% (50.3%)	20%	NR; reported on adherence and convenience of use
	Cluster RCT; 4-5 months; 323/436 (74%)	Family member supervise, daily SMS message to remind taking medication, daily phone call to certify medication	90.8% vs. 93.3%, *p* = NA	NA	88% (52.1%)	74%	NR; reported on adherence, satisfaction, effectiveness, and usability
Phone call							
Liu et al. (25)	RCT; 12 months; 74/74 (100%)	Conduct a follow-up phone call once every 2 weeks	64.86% vs. 59.46%, *p* = 0.632	Comparable	NR (50%)	100%	Reported on high adherence
	RCT; 18 months; 98/98 (100%)	Daily phone call reminds patients taking medication	100% vs. 86%, *p* < 0.001	Effective	3.2% (50%)	NR	Feasible based on adherence and effectiveness

### Quality assessment

Twenty one studies had some concerns regarding bias, and six studies had a high risk of bias. Due to the nature of the trials, blinding was difficult to implement, leading to all studies having some degree or high risk of bias in this area. Additionally, six studies did not report treatment success outcomes, resulting in a high risk of bias. The detailed distribution of bias was shown in Supplementary Figure 1.

### Treatment success

**FIGURE 2:**
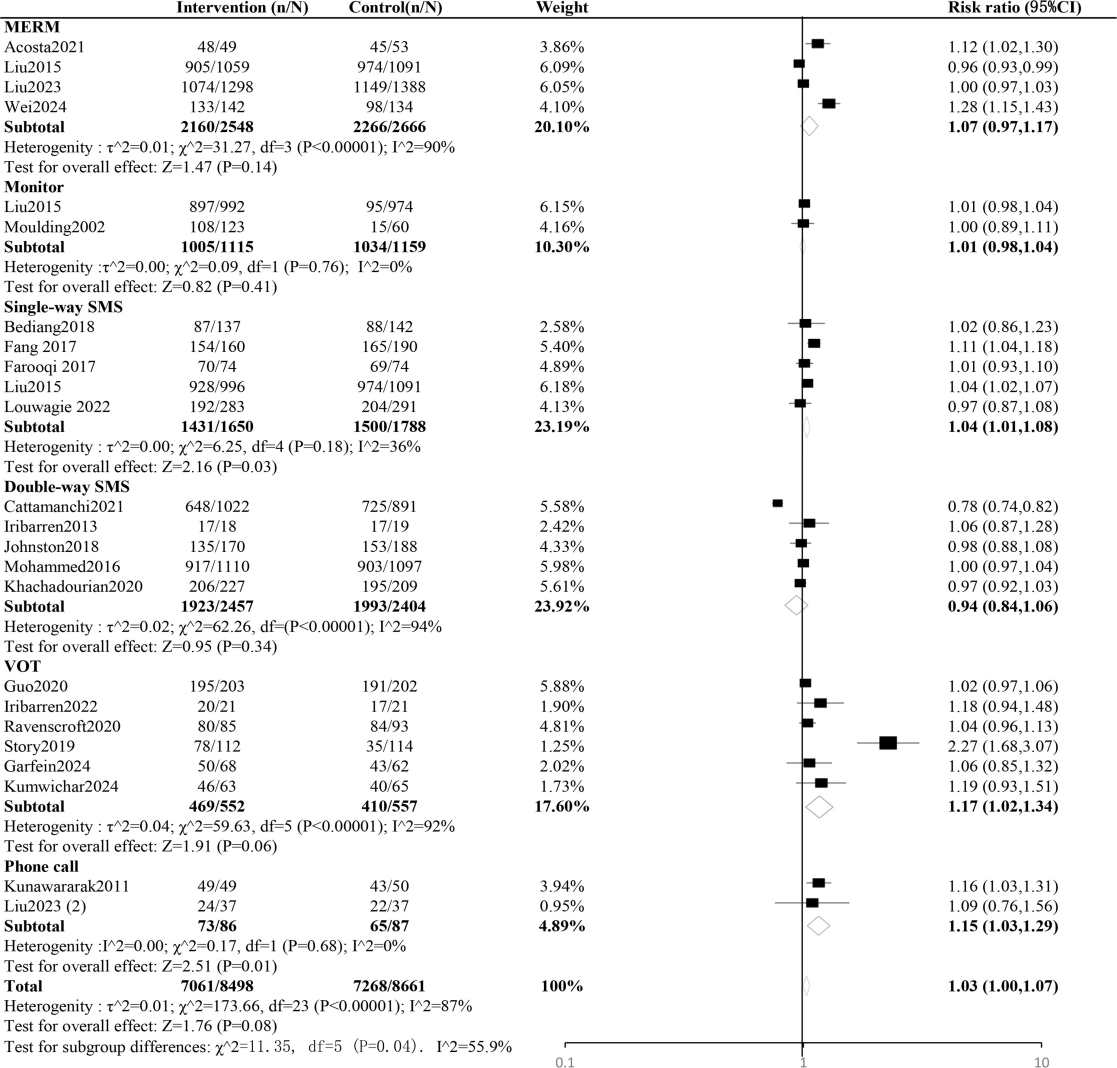
Conventional meta-analysis of treatment success.

A conventional meta-analysis was conducted to explore the overall effect of digital health interventions and the specific impact of each type. Overall, digital health interventions showed significantly higher rate of treatment success than DOT (RR 1.03; 95% CI 1.00–1.07) (Figure 2). As for each type, MERM (RR 1.07; 95% CI 0.97–1.17), monitor (RR 1.01; 95% CI 0.98–1.04), and double-way SMS (RR 0.94; 95% CI 0.84–1.06) did not show a significant statistical difference compared to DOT. However, single-way SMS (RR 1.04; 95% CI 1.01–1.08), VOT (RR 1.17; 95% CI 1.02–1.34), and phone call (RR 1.15; 95% CI 1.03–1.29) demonstrated a significantly higher rate of treatment success than DOT. The quality of evidence ranged from moderate to high. We conducted a subgroup analysis based on the operational model. Since only one study involved real-time VOT and it did not report adherence, we analyzed treatment success. The results showed no significant subgroup effect between the two modalities (*P* = 0.1236) (Supplementary Figure 2).

**FIGURE 3:**
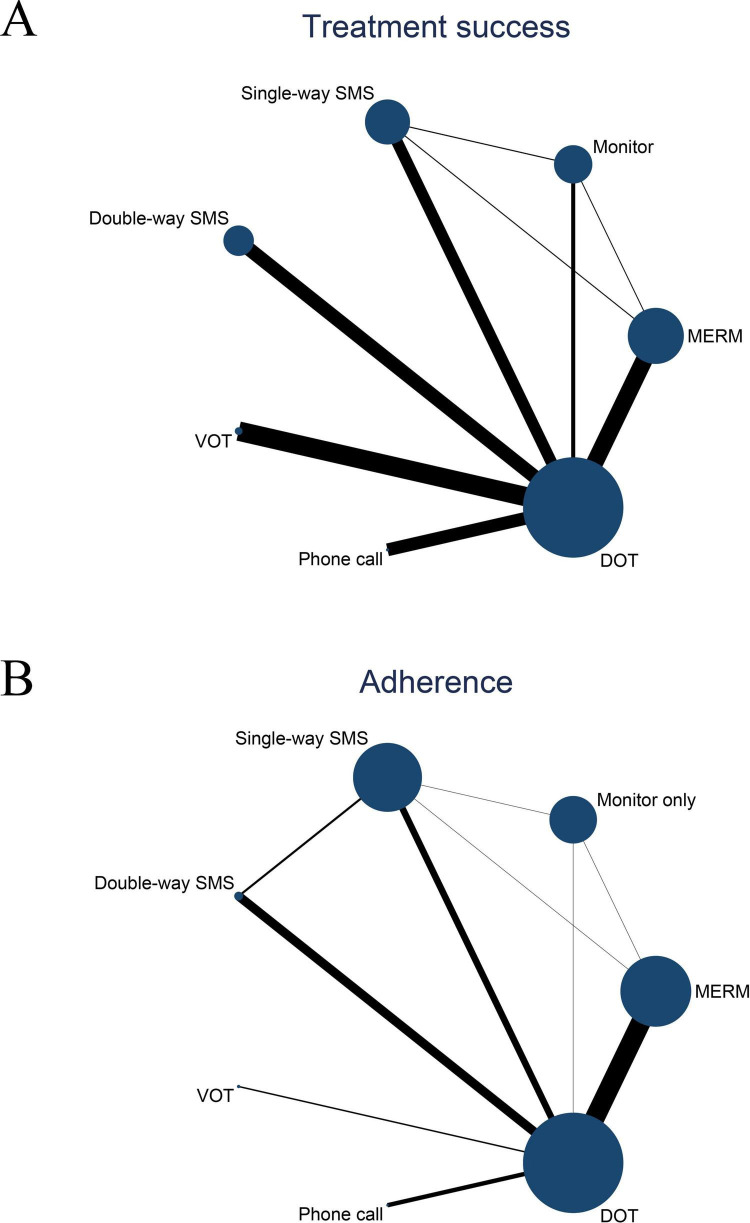
Net-work plot of treatment success **(A)** and adherence **(B)**.

**FIGURE 4:**
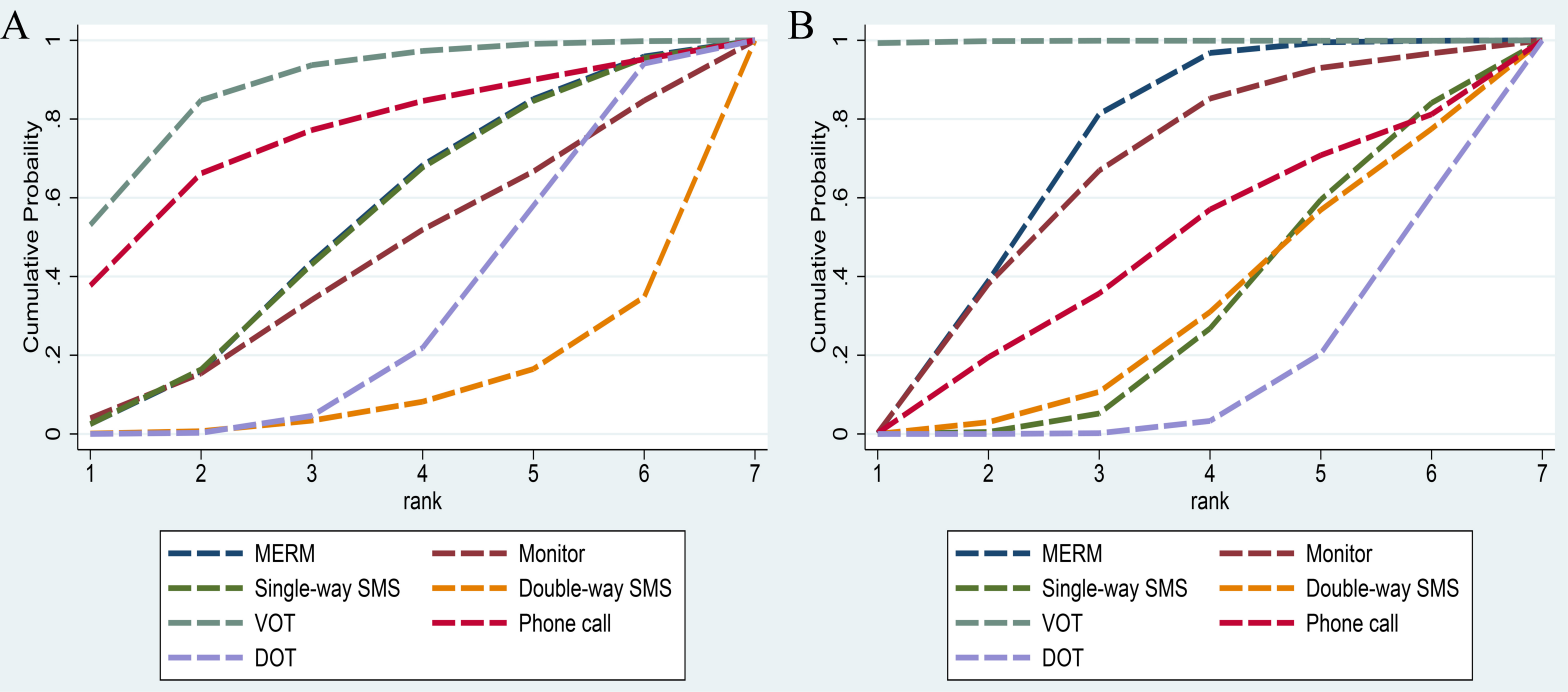
The cumulative probability of first rank of treatment success **(A)** and adherence **(B)**.

The network analysis of the comparative outcomes of digital health interventions on treatment success was presented in Figure 3A (the thickness of black line segments indicates the number of comparative studies, and blue dots represent the sample size). A total of 23 studies reported treatment success, involving 7 different interventions. Among these, VOT had the largest sample size after DOT, followed by MERM. A net-league comparing the RR and 95% CI of these digital health interventions revealed no statistically significant differences among them, except that VOT showed significantly higher treatment success compared to DOT (RR 1.18; 95% CI 1.02–1.37) and double-way SMS showed significantly lower rate of treatment success than VOT (RR 0.80; 95% CI 0.67–0.95) (Supplementary Table 1). The best-ranked probability sorted by SUCRA, are as follows: VOT (SUCRA 88%), phone call (SUCRA 75.1%), single-way SMS (SUCRA 51.9%), MERM (SUCRA 51.6%), monitor (SUCRA 43.4%), DOT (SUCRA 29.4%), and double-way SMS (SUCRA 9.8%). The cumulative probability of rank was showed in Figure 4A. Based on these findings, VOT appeared to be a promising intervention for achieving better treatment success.

### Adherence

The network analysis of the comparative outcomes of digital health interventions on adherence was presented in Figure 3B. The net-league showed that compared to VOT, other digital health intervention and DOT had significantly lower adherence, MERM also showed significantly higher adherence than DOT (Supplementary Table 2). The best-ranked probability sorted by SUCRA, are as follows: VOT (SUCRA 99%), MERM (SUCRA 69.5%), monitor (SUCRA 63.4%), phone call (SUCRA 44%), double-way SMS (SUCRA 29.5%), single-way SMS (SUCRA 29.3%), DOT (SUCRA 14.1%). The cumulative probability of rank was showed in Figure 4B. Based on these findings, VOT appeared to be a promising intervention for achieving better adherence.

### Loss to follow-up

The network analysis of the comparative outcomes of digital health interventions on loss to follow-up was presented in Supplementary Figure 3. The net-league showed that there was no statistically significant between each intervention. The best-ranked probability sorted by SUCRA, are as follows: VOT (SUCRA 84.9%), single-way SMS (SUCRA 68.2%), monitor (SUCRA 53.1%), MERM (SUCRA 51%), double-way SMS (SUCRA 26.3%), DOT (SUCRA 16.5%). The cumulative probability of rank was showed in Supplementary Figure 4. Although there were no significant statistical differences between interventions, the best-ranked probability suggests that VOT has a better advantage in reducing loss to follow-up.

Four studies also reported the characteristic of participants who lost to follow-up or having low adherence. It found that participants with higher level education tend to compliance with medication. However, considering the uneven distribution of participants number in each domain, caution was need to explain these results.

### Heterogeneity, consistency, sensitivity analysis, and publication bias

The I^2^ of treatment success and adherence were 7 and 10%, which showed no significant heterogeneity.

The node-splitting method was used to assess the local consistency of treatment success and adherence. In the analysis of treatment success, there were three loops and the results showed consistency (all *P* > 0.05). In the analysis of adherence, there were two loops and the results showed consistency (all *P* > 0.05).

Sensitivity analysis was conducted on treatment success and adherence by using both consistency model and inconsistency model. As for best-ranked probability, VOT still hold the most promising place in both models.

The funnel plot was used to assess publication bias for treatment success (Supplementary Figure 5). The colored dots represent various comparisons between different intervention measures, primarily concentrated in the upper-middle section. We observed no significant asymmetry in the adjusted comparison funnel plot, suggesting that publication bias in our study is negligible.

### GRADE assessment

For treatment success, all interventions, including monitor and reminder, monitor alone, single-way SMS, double-way SMS, VOT, and phone call, were rated as having very low certainty of evidence. The main reason for downgrading was major concerns regarding imprecision, while reporting bias was also judged to be of some concern across interventions. For adherence, the certainty of evidence was similarly rated as very low for most interventions, including monitor and reminder, monitor alone, single-way SMS, double-way SMS, and phone call, primarily due to major concerns about imprecision and, to a lesser extent, reporting bias. Notably, VOT was the only intervention with a higher rating, achieving moderate certainty of evidence, as it showed no major concerns across the evaluated domains. For lost to follow-up, all interventions were rated as having very low certainty of evidence. The main reason for downgrading was major concerns regarding imprecision (Supplementary Table 3).

## Discussion

This study included 29 randomized controlled trials and is the first network meta-analysis to explore the effectiveness of various digital health interventions in tuberculosis treatment. We analyzed treatment success and adherence based on the WHO’s definitions of tuberculosis treatment outcomes. The results showed that, overall, digital health interventions helped improve treatment success, but some did not show significant advantages compared to DOT. In the network meta-analysis, we found that demonstrated promising effects on both treatment success and adherence. Our subgroup analysis by VOT operational mode did not reveal significant differences in treatment success between synchronous (real-time) and asynchronous (recorded) modalities. The lack of significant subgroup effect may be partly due to the limited number of studies using real-time VOT, as only one study was available and adherence data were not reported. These findings suggest that, based on the current evidence, both synchronous and asynchronous VOT can achieve comparable treatment success, although further studies with larger sample sizes and adherence reporting are needed to confirm this.

In 2018, Alipanah et al. conducted a traditional meta-analysis that included 129 studies (29% RCTs and 71% observational studies) exploring interventions such as social support, supervised treatment, and digital health interventions to promote treatment adherence (16). Only three RCTs focused on the digital health intervention. The results showed that TB treatment outcomes improved with adherence interventions like patient education and counseling, incentives and enablers, psychological interventions, reminders and tracers, and digital health technologies. Trained healthcare providers and community delivery offered patient-centered DOT options that enhanced adherence and improved treatment outcomes compared to unsupervised SAT alone. Our study used WHO-recommended DOT as a control and included only randomized controlled trials, specifically conducting a network meta-analysis on existing digital health interventions, including medication event reminder monitor systems, SMS, monitors, video observed therapy, and phone calls. The results found, similar to previous findings, that digital health improved medication adherence in tuberculosis patients, with video observed therapy particularly enhancing treatment success and adherence. Compared with the network meta-analysis by Cheng et al. (35), which included 27 RCTs and established hierarchies of digital health interventions for tuberculosis treatment, our study incorporated 29 RCTs with 17,800 participants up to May 2026, thus including more recent evidence. We further refined the classification of interventions (e.g., one-way/two-way SMS, VOT, MERM, phone calls, monitoring systems), analyzed differences between treatment success and adherence, evaluated loss to follow-up and sensitivity analyses, and explored the impact of participant characteristics such as educational level and low-adherence populations. Therefore, our study not only updates the existing evidence but also provides more specific and clinically actionable guidance, representing a clear incremental contribution to the literature.

Incomplete adherence to tuberculosis treatment increased the risk of delayed sputum culture conversion, community transmission, treatment failure, relapse, and the development or amplification of drug resistance (36). Therefore, developing effective strategies to improve patient adherence was crucial for optimal treatment outcomes. With advancements in digital health technologies, various approaches had been applied to enhance tuberculosis treatment adherence. In a conventional meta-analysis comparing video observed therapy versus DOT, it was found that the implementation of video observed therapy significantly enhanced medication adherence and bacteriological resolution in tuberculosis-infected patients compared to DOT (37). Our findings indicated that video observed therapy was particularly effective among these digital interventions. This method used video technology to monitor patients taking their medications, allowing doctors or nurses to ensure that patients took their medications on time and correctly. In our study, we found that two-way SMS was not as effective as anticipated, and in some cases, its outcomes were inferior to one-way SMS. We speculated that in instances where two-way SMS was less effective, some patients might have falsely reported taking their medications. This could have been because responding with additional messages or calls was cumbersome, making it easier for patients to lie, which was difficult to verify. Future research on digital health interventions should consider the convenience for patients, potential disruptions to daily life, and the ability of healthcare workers to accurately assess patient adherence. Our network meta-analysis is a comprehensive and systematic study of digital health interventions for tuberculosis treatment, incorporating only high-quality randomized controlled trials. This approach ensures the accuracy and validity of our findings, providing a more extensive overview of current evidence compared to traditional meta-analyses. As a result, healthcare professionals can make better-informed decisions regarding the most effective interventions for tuberculosis patients.

However, our study has some limitations. Firstly, due to the challenges in conducting randomized controlled trials in this field, certain interventions were represented by fewer studies, highlighting the need for further research and updates. Secondly, although we used a random effects model and found no significant statistical heterogeneity between studies, actual heterogeneity cannot be overlooked. Differences in the content and timing of single-way SMS messages, for example, may affect the reliability of the results. Thirdly, we concentrated only on primary or representative outcome measures, and other indicators such as recurrence and drug resistance development were not analyzed due to limited reporting. Fourthly, we were unable to assess whether adherence matched actual medication intake, which could lead to discrepancies between adherence and treatment success. Fifthly, drug-resistant tuberculosis remains a significant challenge in treatment. Since the studies included did not specifically address this issue, we could not evaluate the effects of digital health interventions on drug-resistant tuberculosis. Sixthly, we did not perform a cost-effectiveness analysis; although video observed therapy (VOT) appeared most effective, its cost-effectiveness in different settings remains to be determined. Finally, some included trials had challenges with blinding and adherence measurement, which may lead to potential overestimation of effect sizes. These limitations should be considered when interpreting our results, and further research is necessary to explore strategies for improving adherence, including in drug-resistant tuberculosis and resource-limited settings. Future research could focus on increasing the number of RCTs for underrepresented interventions such as monitor-based systems, phone calls, and two-way SMS, conducting cost-effectiveness analyses, and incorporating objective compliance verification to reduce measurement bias and improve adherence assessment.

## Conclusion

Overall, digital health interventions can improve the treatment outcomes of tuberculosis medication. Among digital health interventions, video observed therapy has shown effectiveness for treatment success and adherence in patients with tuberculosis compared to other digital health interventions. However, our study included a limited number of studies. Future research is needed to further explore the personalized application of digital health interventions for different tuberculosis patients.

## Data Availability

The original contributions presented in this study are included in the article/Supplementary material, further inquiries can be directed to the corresponding author.

## References

[1] World Health Organiztion [WHO] (2025). ijo

[2] Uchmanowicz B Jankowska EA Uchmanowicz I Morisky DE. Self-reported medication adherence measured with morisky medication adherence scales and its determinants in hypertensive patients aged ≥60 Years: a systematic review and meta-analysis. *Front Pharmacol*. (2019) 10:168. 10.3389/fphar.2019.00168 30930769 PMC6425867

[3] Alsayed SSR Gunosewoyo H. Tuberculosis: pathogenesis, current treatment regimens and new drug targets. *Int J Mol Sci*. (2023) 24:5202. 10.3390/ijms24065202 36982277 PMC10049048

[4] Nezenega ZS Perimal-Lewis L Maeder AJ. Factors influencing patient adherence to tuberculosis treatment in ethiopia: a literature review. *Int J Environ Res Public Health*. (2020) 17:5626. 10.3390/ijerph17155626 32759876 PMC7432798

[5] Yin J Yuan J Hu Y Wei X. Association between directly observed therapy and treatment outcomes in multidrug-resistant tuberculosis: a systematic review and meta-analysis. *PLoS One*. (2016) 11:e0150511. 10.1371/journal.pone.0150511 26930287 PMC4773051

[6] Lin Q Ma W Xu M Xu Z Wang J Liang Z et al. A clinical prognostic model related to T cells based on machine learning for predicting the prognosis and immune response of ovarian cancer. *Heliyon*. (2024) 10:e36898. 10.1016/j.heliyon.2024.e36898 39296051 PMC11409031

[7] Wang H Fan M Wu J Liu H. Development of a questionnaire on supportive care needs of patients with COPD combined with respiratory failure on noninvasive ventilators and evaluating its reliability and validity. *Respir Med*. (2025) 248:108384. 10.1016/j.rmed.2025.108384 41022227

[8] Karumbi J Garner P. Directly observed therapy for treating tuberculosis. *Cochrane Database Syst Rev*. (2015) 2015:CD003343. 10.1002/14651858.CD003343.pub4 26022367 PMC4460720

[9] Yu H Lin C Chen F Chen L Liu W Liu H et al. Dose response test of patient-derived cancer organoids to irradiation. *Front Oncol*. (2025) 15:1677172. 10.3389/fonc.2025.1677172 41220945 PMC12597761

[10] Yu Z Li J He Z Li W Lian A Xia Y et al. PGC-1α mediates hypoxia-preconditioned olfactory mucosa mesenchymal stem cells improved neuroinflammatory response via inhibiting microglial ferroptosis in ischemic stroke. *J Transl Med*. (2025) 23:1263. 10.1186/s12967-025-07240-5 41219768 PMC12606990

[11] Moschonis G Siopis G Jung J Eweka E Willems R Kwasnicka D et al. Effectiveness, reach, uptake, and feasibility of digital health interventions for adults with type 2 diabetes: a systematic review and meta-analysis of randomised controlled trials. *Lancet Digit Health*. (2023) 5:e125–43. 10.1016/S2589-7500(22)00233-3 36828606

[12] Siopis G Moschonis G Eweka E Jung J Kwasnicka D Asare BY et al. Effectiveness, reach, uptake, and feasibility of digital health interventions for adults with hypertension: a systematic review and meta-analysis of randomised controlled trials. *Lancet Digit Health*. (2023) 5:e144–59. 10.1016/S2589-7500(23)00002-X 36828607

[13] Cao Z Zhu J Wang Z Peng Y Zeng L. Comprehensive pan-cancer analysis reveals ENC1 as a promising prognostic biomarker for tumor microenvironment and therapeutic responses. *Sci Rep*. (2024) 14:25331. 10.1038/s41598-024-76798-9 39455818 PMC11512054

[14] Huang C Ding Y Xu S Chen R Jiang T Zeng B et al. Causal associations of self-reported walking pace with respiratory diseases: a Mendelian randomization analysis. *Medicine*. (2025) 104:e41746. 10.1097/MD.0000000000041746 40101097 PMC11922406

[15] Lee S Rajaguru V Baek JS Shin J Park Y. Digital health interventions to enhance tuberculosis treatment adherence: scoping review. *JMIR Mhealth Uhealth*. (2023) 11:e49741. 10.2196/49741 38054471 PMC10718480

[16] Alipanah N Jarlsberg L Miller C Linh NN Falzon D Jaramillo E et al. Adherence interventions and outcomes of tuberculosis treatment: a systematic review and meta-analysis of trials and observational studies. *PLoS Med*. (2018) 15:e1002595. 10.1371/journal.pmed.1002595 29969463 PMC6029765

[17] Glasgow RE Harden SM Gaglio B Rabin B Smith ML Porter GC et al. RE-AIM planning and evaluation framework: adapting to new science and practice with a 20-year review. *Front Public Health*. (2019) 7:64. 10.3389/fpubh.2019.00064 30984733 PMC6450067

[18] Moore GF Audrey S Barker M Bond L Bonell C Hardeman W et al. Process evaluation of complex interventions: medical research council guidance. *BMJ*. (2015) 350:h1258. 10.1136/bmj.h1258 25791983 PMC4366184

[19] Skivington K Matthews L Simpson SA Craig P Baird J Blazeby JM et al. A new framework for developing and evaluating complex interventions: update of Medical Research Council guidance. *BMJ*. (2021) 374:n2061. 10.1136/bmj.n2061 34593508 PMC8482308

[20] Balshem H Helfand M Schünemann HJ Oxman AD Kunz R Brozek J et al. GRADE guidelines: 3. Rating the quality of evidence. *J Clin Epidemiol*. (2011) 64:401–6. 10.1016/j.jclinepi.2010.07.015 21208779

[21] Farooqi RJ Ashraf S Zaman M. The role of mobile SMS-reminders in improving drugs compliance in patients receiving anti-TB treatment from DOTS program. *J Postgraduate Med Inst.* (2017) 31:156–62.

[22] Wei X Hicks JP Zhang Z Haldane V Pasang P Li L et al. Effectiveness of a comprehensive package based on electronic medication monitors at improving treatment outcomes among tuberculosis patients in Tibet: a multicentre randomised controlled trial. *Lancet*. (2024) 403:913–23. 10.1016/S0140-6736(23)02270-5 38309280

[23] Musiimenta A Tumuhimbise W Atukunda EC Mugaba AT Musinguzi N Muzoora C et al. The feasibility, acceptability, and preliminary impact of real-time monitors and SMS on tuberculosis medication adherence in southwestern Uganda: findings from a mixed methods pilot randomized controlled trial. *PLoS Glob Public Health*. (2023) 3:e0001813. 10.1371/journal.pgph.0001813 38051699 PMC10697590

[24] Liu X Thompson J Dong H Sweeney S Li X Yuan Y et al. Digital adherence technologies to improve tuberculosis treatment outcomes in China: a cluster-randomised superiority trial. *Lancet Glob Health*. (2023) 11:e693–703. 10.1016/S2214-109X(23)00068-2 37061308 PMC10126227

[25] Liu A Yin X Wang R Cheng L Zhang Y Tu F et al. Analysis of the effect of anti-tuberculosis therapy combined with all-in-one nursing care on the alleviation of inflammation in patients with pulmonary tuberculosis. *Cell Mol Biol*. (2023) 69:131–6. 10.14715/cmb/2022.69.1.23 37213145

[26] Manyazewal T Woldeamanuel Y Holland DP Fekadu A Marconi VC. Effectiveness of a digital medication event reminder and monitor device for patients with tuberculosis (SELFTB): a multicenter randomized controlled trial. *BMC Med*. (2022) 20:310. 10.1186/s12916-022-02521-y 36167528 PMC9514884

[27] Louwagie G Kanaan M Morojele NK Van Zyl A Moriarty AS Li J et al. Effect of a brief motivational interview and text message intervention targeting tobacco smoking, alcohol use and medication adherence to improve tuberculosis treatment outcomes in adult patients with tuberculosis: a multicentre, randomised controlled trial of the ProLife programme in South Africa. *BMJ Open*. (2022) 12:e056496. 10.1136/bmjopen-2021-056496 35165113 PMC8845202

[28] Iribarren SJ Milligan H Chirico C Goodwin K Schnall R Telles H et al. Patient-centered mobile tuberculosis treatment support tools (TB-TSTs) to improve treatment adherence: a pilot randomized controlled trial exploring feasibility, acceptability and refinement needs. *Lancet Reg Health Am*. (2022) 13:100291. 10.1016/j.lana.2022.100291 36061038 PMC9426680

[29] Acosta J Flores P Alarcón M Grande-Ortiz M Moreno-Exebio L Puyen ZM. A randomised controlled trial to evaluate a medication monitoring system for TB treatment. *Int J Tuberc Lung Dis*. (2022) 26:44–9. 10.5588/ijtld.21.0373 34969428 PMC8734191

[30] Cattamanchi A Crowder R Kityamuwesi A Kiwanuka N Lamunu M Namale C et al. Digital adherence technology for tuberculosis treatment supervision: a stepped-wedge cluster-randomized trial in Uganda. *PLoS Med*. (2021) 18:e1003628. 10.1371/journal.pmed.1003628 33956802 PMC8136841

[31] Ravenscroft L Kettle S Persian R Ruda S Severin L Doltu S et al. Video-observed therapy and medication adherence for tuberculosis patients: randomised controlled trial in Moldova. *Eur Respir J*. (2020) 56:2000493. 10.1183/13993003.00493-2020 32381495

[32] Johnston JC van der Kop ML Smillie K Ogilvie G Marra F Sadatsafavi M et al. The effect of text messaging on latent tuberculosis treatment adherence: a randomised controlled trial. *Eur Respir J.* (2018) 51. 10.1183/13993003.01488-2017 29437940

[33] Liu X Lewis JJ Zhang H Lu W Zhang S Zheng G et al. Effectiveness of electronic reminders to improve medication adherence in tuberculosis patients: a cluster-randomised trial. *PLoS Med*. (2015) 12:e1001876. 10.1371/journal.pmed.1001876 26372470 PMC4570796

[34] Kibu OD Siysi VV Albert Legrand SE Asangbeng Tanue E Nsagha DS. Treatment adherence among HIV and TB patients using single and double way mobile phone text messages: a randomized controlled trial. *J Trop Med*. (2022) 2022:2980141. 10.1155/2022/2980141 35996467 PMC9392638

[35] Cheng Q Chen P Dai R Jia Q Bai X Cao Q et al. Comparative effectiveness of digital health technologies in tuberculosis treatment: systematic review and network meta-analysis of randomized controlled trials. *JMIR Mhealth Uhealth*. (2025) 13:e75424. 10.2196/75424 40957002 PMC12440258

[36] Leddy AM Jaganath D Triasih R Wobudeya E Bellotti de Oliveira MC Sheremeta Y et al. Social determinants of adherence to treatment for tuberculosis infection and disease among children, adolescents, and young adults: a narrative review. *J Pediatric Infect Dis Soc*. (2022) 11:S79–84. 10.1093/jpids/piac058 36314549 PMC9620428

[37] Truong CB Tanni KA Qian J. Video-observed therapy versus directly observed therapy in patients with tuberculosis. *Am J Prev Med*. (2022) 62:450–8. 10.1016/j.amepre.2021.10.013 34916094

