# Supplementary material for: The role of digital health interventions for adults with tuberculosis: a network meta-analysis of randomized controlled trials
Source: Front Med (Lausanne). 2026 Jun 9;13:1840258. doi: 10.3389/fmed.2026.1840258 (PMC13286891; doi:10.3389/fmed.2026.1840258)
Supplement: Supplementary file 2 [file Table_1.DOCX]

**Supplement**

[Search strategy 2](#_Toc230107775)

[eFigure1. Risk of bias of included studies 4](#_Toc230107776)

[eFigure2. Subgroup analysis on treatment success by VOT operational model 5](#_Toc230107777)

[eFigure3. Plotwork of loss to follow-up 5](#_Toc230107778)

[eFigure4. The cumulative probability of rank in loss to follow-up 6](#_Toc230107779)

[eTable1. Net-league table of treatment success 7](#_Toc230107780)

[eTable2. Net-league table of adherence 8](#_Toc230107781)

[eFigure5. Publication bias 9](#_Toc230107782)

[eTable3. Grade of result for conventional meta-analysis 10](#_Toc230107783)

# Search strategy

PubMed

1. "Tuberculosis"[Mesh] OR “Tuberculosis”[tiab] OR “Tuberculoses”[tiab]

2. “mHealth”[Tiab] OR “mobile Health”[Tiab] OR “health app*”[Tiab] OR “digital health”[Tiab] OR “online health”[Tiab] OR “app”[Tiab] OR “application”[tiab] OR “phone”[Tiab] OR “smartphone”[Tiab] OR “SMS”[Tiab] OR “message”[Tiab] OR “web site”[Tiab] OR “digital”[Tiab]

3. “medication” [tiab] OR “drug”[tiab] OR “adherence”[tiab] OR “compliance”[tiab]

3.#1 AND #2 AND #3

4. ((compar*[tiab]) OR ((singl*[tiab] OR doubl*[tiab] OR tripl*[tiab]) and (mask*[tiab] OR blind*[tiab]))) OR (random*[tiab] OR placebo[tiab] OR controlled[tiab] OR trial*[tiab])

5. #3 AND #4

Cochrane Library

1. MeSH descriptor: [Tuberculosis] explode all trees

2. (Tuberculosis OR Tuberculoses):ti,ab

3. #1 OR #2

4. (mHealth OR mobile Health OR health app* OR digital health OR online health OR app OR application OR phone OR smartphone OR SMS OR message OR web site OR digital):ti,ab

5. (medication or drug or adherence or compliance):ti,ab

6. #3 AND #4 AND #5

7. ((compar*) OR ((singl* or doubl* or tripl*) and (mask* or blind*))) OR (random* or placebo or controlled or trial*):ti,ab

8. #6 AND #7

Embase

1. 'tuberculosis'/exp

2. ((tuberculosis) OR (tuberculoses)):ti,ab

3. #1 OR #2

4. ((mHealth) OR (mobile Health) OR (health app*) OR (digital health) OR (online health) OR (app) OR (application) OR (phone) OR (smartphone) OR (SMS) OR (message) OR (web site) OR (digital)):ti,ab

5. ((medication) or (drug) or (adherence) or (compliance)):ti,ab

6. #3 AND #4

7. compar* OR ((singl* OR doubl* OR tripl*) AND (mask* OR blind*)) OR random*:ti,ab OR placebo:ti,ab OR controlled:ti,ab OR trial*:ti,ab

8. #5 AND #6

Web of science

1. TS=(Tuberculosis OR Tuberculoses)

2. TS=(mHealth OR mobile Health OR health app* OR digital health OR online health OR app OR application OR phone OR smartphone OR SMS OR message OR web site OR digital)

3. TS=(medication or drug or adherence or compliance)

4. #1 AND #2 AND #3

5. TS=(((compar*) OR ((singl* or doubl* or tripl*) and (mask* or blind*))) OR (random* or placebo or controlled or trial*))

6. #4 AND #5

# eFigure1. Risk of bias of included studies


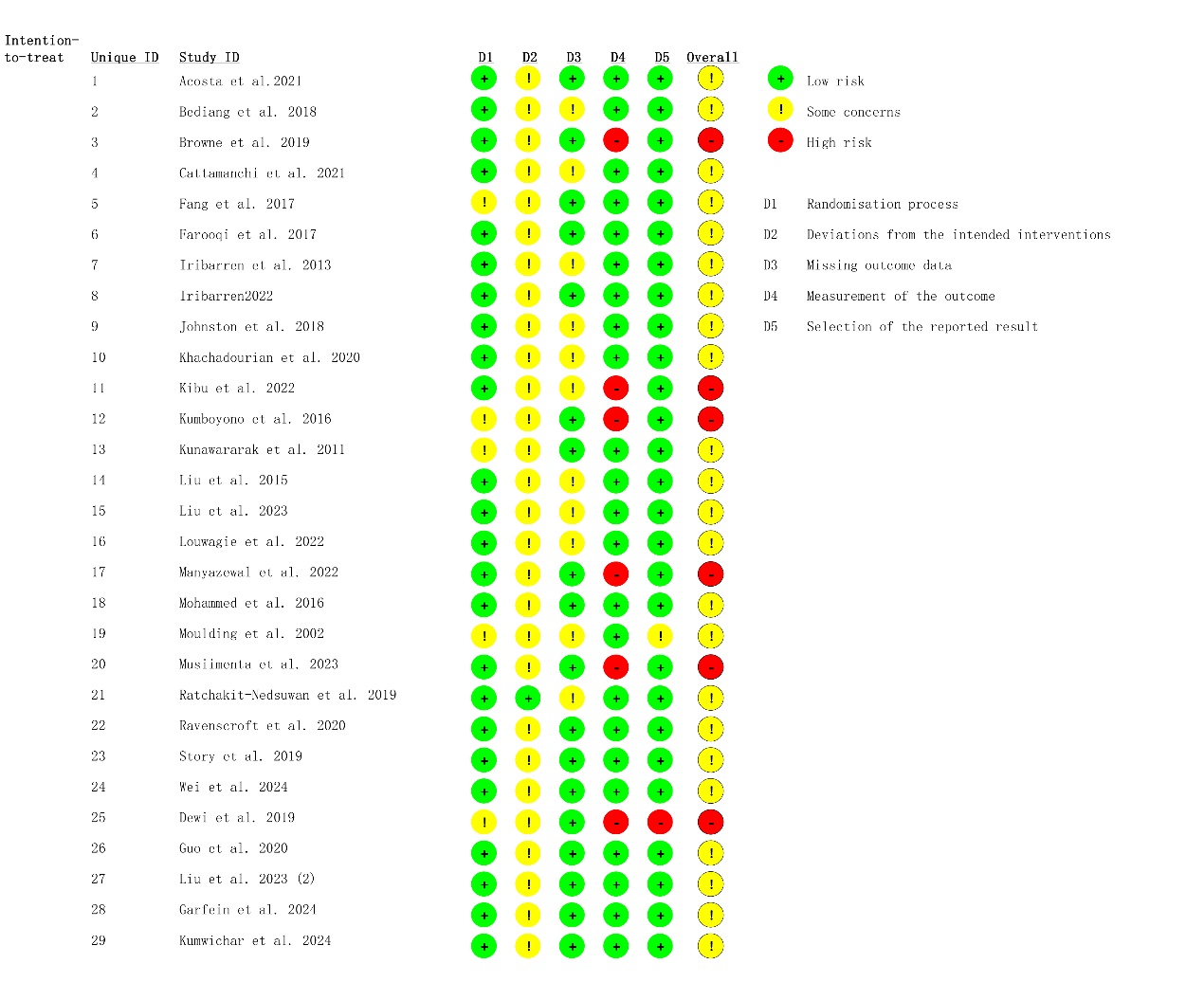


## eFigure2. Subgroup analysis on treatment success by VOT operational model

**
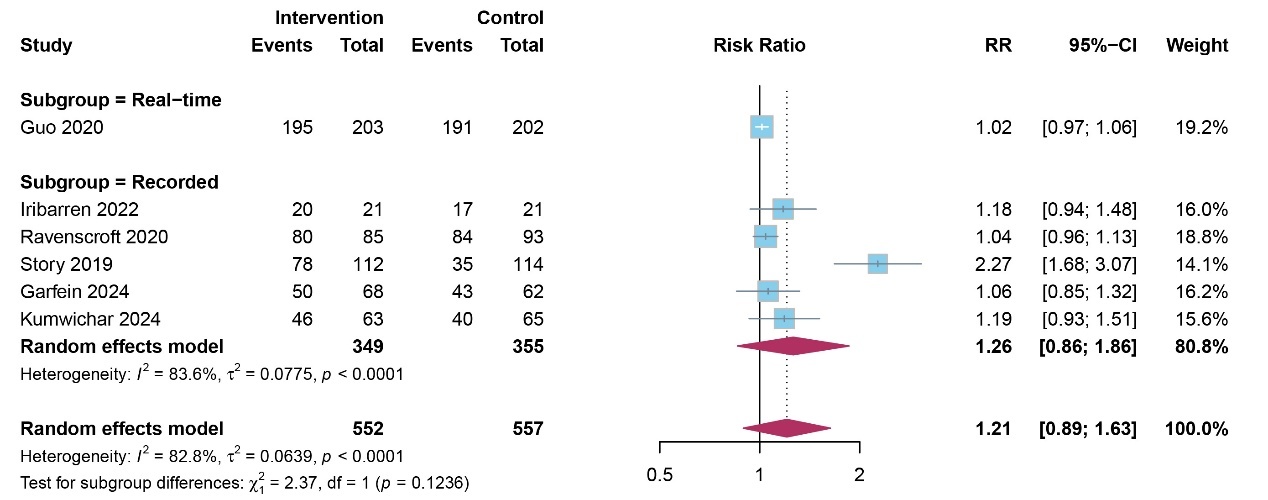
**

# eFigure3. Plotwork of loss to follow-up

**
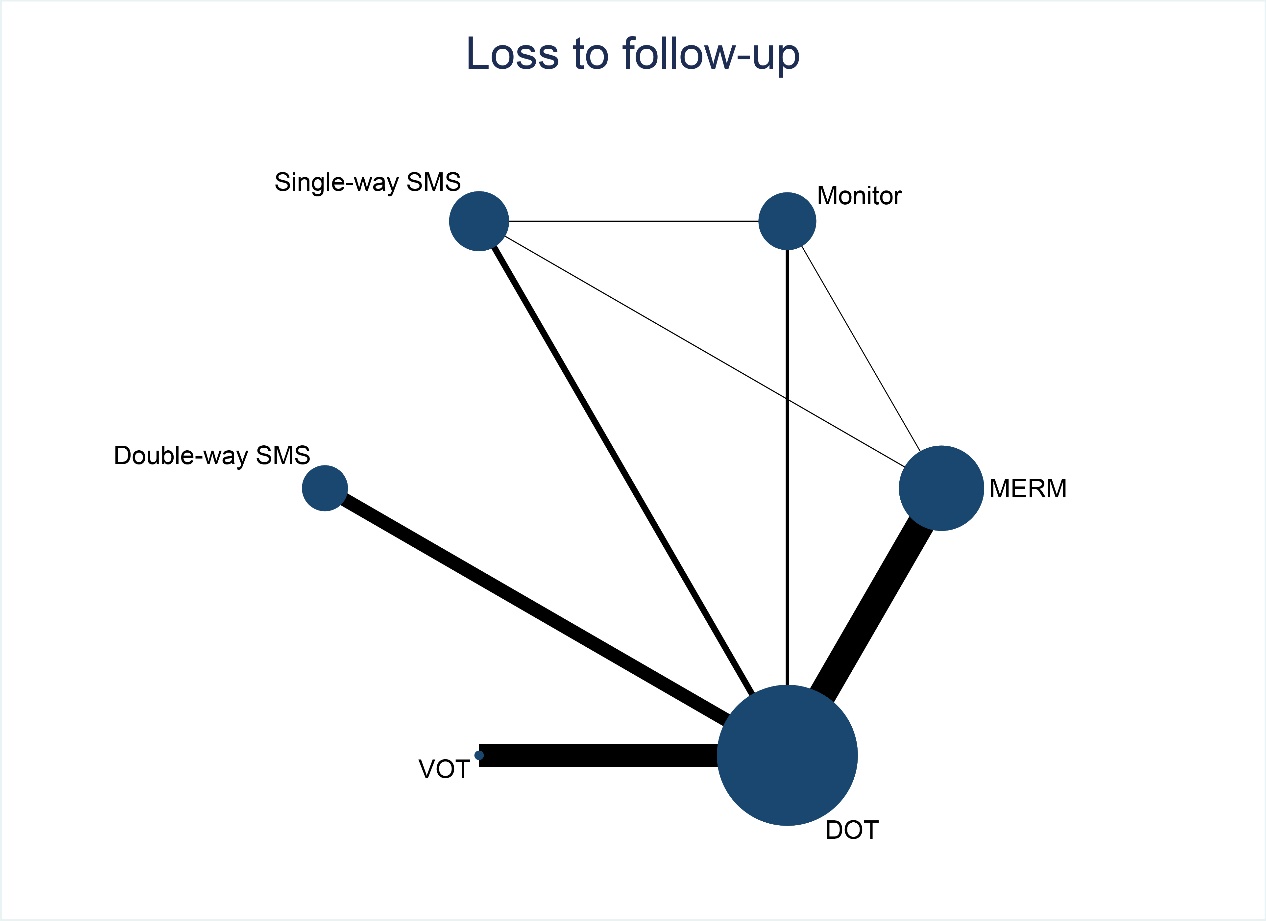
**

# eFigure4. The cumulative probability of rank in loss to follow-up

**
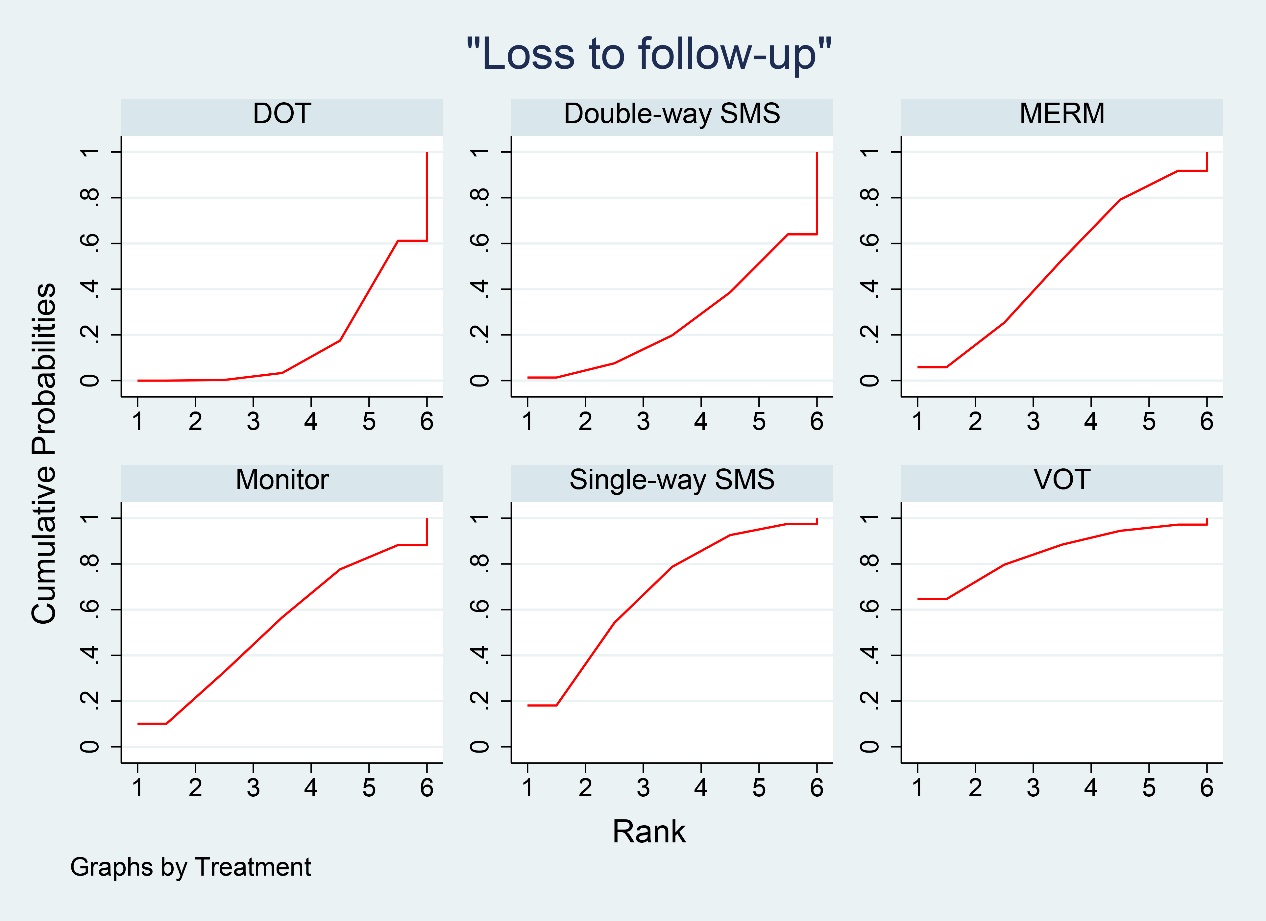
**

# eTable1. Net-league table of treatment success

| MERM | 0.98 (0.82,1.17) | 1.00 (0.87,1.15) | 0.90 (0.77,1.06) | 1.13 (0.94,1.36) | 1.09 (0.85,1.40) | 0.96 (0.86,1.07) |
| --- | --- | --- | --- | --- | --- | --- |
| 1.02 (0.85,1.22) | Monitor | 1.02 (0.85,1.22) | 0.92 (0.76,1.12) | 1.15 (0.93,1.43) | 1.11 (0.85,1.46) | 0.97 (0.83,1.14) |
| 1.00 (0.87,1.15) | 0.98 (0.82,1.17) | Single-way SMS | 0.90 (0.77,1.06) | 1.13 (0.94,1.36) | 1.09 (0.85,1.40) | 0.96 (0.86,1.07) |
| 1.11 (0.95,1.29) | 1.09 (0.89,1.32) | 1.11 (0.95,1.30) | Double-way SMS | 1.25 (1.04,1.51) | 1.21 (0.94,1.55) | 1.06 (0.95,1.19) |
| 0.88 (0.74,1.06) | 0.87 (0.70,1.08) | 0.88 (0.73,1.07) | 0.80 (0.66,0.96) | VOT | 0.97 (0.74,1.27) | 0.85 (0.73,0.98) |
| 0.92 (0.71,1.17) | 0.90 (0.68,1.18) | 0.92 (0.71,1.17) | 0.83 (0.64,1.06) | 1.04 (0.79,1.36) | Phone call | 0.88 (0.70,1.10) |
| 1.05 (0.94,1.16) | 1.03 (0.88,1.20) | 1.05 (0.94,1.17) | 0.94 (0.84,1.06) | 1.18 (1.02,1.37) | 1.14 (0.91,1.43) | DOT |

# eTable2. Net-league table of adherence

| MERM | 0.98 (0.65,1.47) | 0.77 (0.58,1.02) | 0.77 (0.54,1.10) | 2.72 (1.40,5.28) | 0.85 (0.50,1.45) | 0.71 (0.58,0.87) |
| --- | --- | --- | --- | --- | --- | --- |
| 1.02 (0.68,1.54) | Monitor | 0.78 (0.52,1.18) | 0.78 (0.48,1.28) | 2.78 (1.32,5.85) | 0.87 (0.46,1.63) | 0.72 (0.49,1.08) |
| 1.30 (0.98,1.73) | 1.28 (0.85,1.93) | Single-way SMS | 1.00 (0.70,1.43) | 3.54 (1.81,6.93) | 1.11 (0.65,1.90) | 0.93 (0.74,1.16) |
| 1.30 (0.91,1.86) | 1.27 (0.78,2.08) | 1.00 (0.70,1.42) | Double-way SMS | 3.54 (1.76,7.11) | 1.11 (0.63,1.96) | 0.92 (0.69,1.24) |
| 0.37 (0.19,0.72) | 0.36 (0.17,0.76) | 0.28 (0.14,0.55) | 0.28 (0.14,0.57) | VOT | 0.31 (0.14,0.70) | 0.26 (0.14,0.49) |
| 1.17 (0.69,2.00) | 1.15 (0.61,2.16) | 0.90 (0.53,1.54) | 0.90 (0.51,1.60) | 3.19 (1.43,7.10) | Phone call | 0.83 (0.51,1.36) |
| 1.41 (1.15,1.73) | 1.38 (0.93,2.05) | 1.08 (0.86,1.35) | 1.08 (0.80,1.46) | 3.83 (2.03,7.21) | 1.20 (0.74,1.96) | DOT |

# eFigure5. Publication bias

**
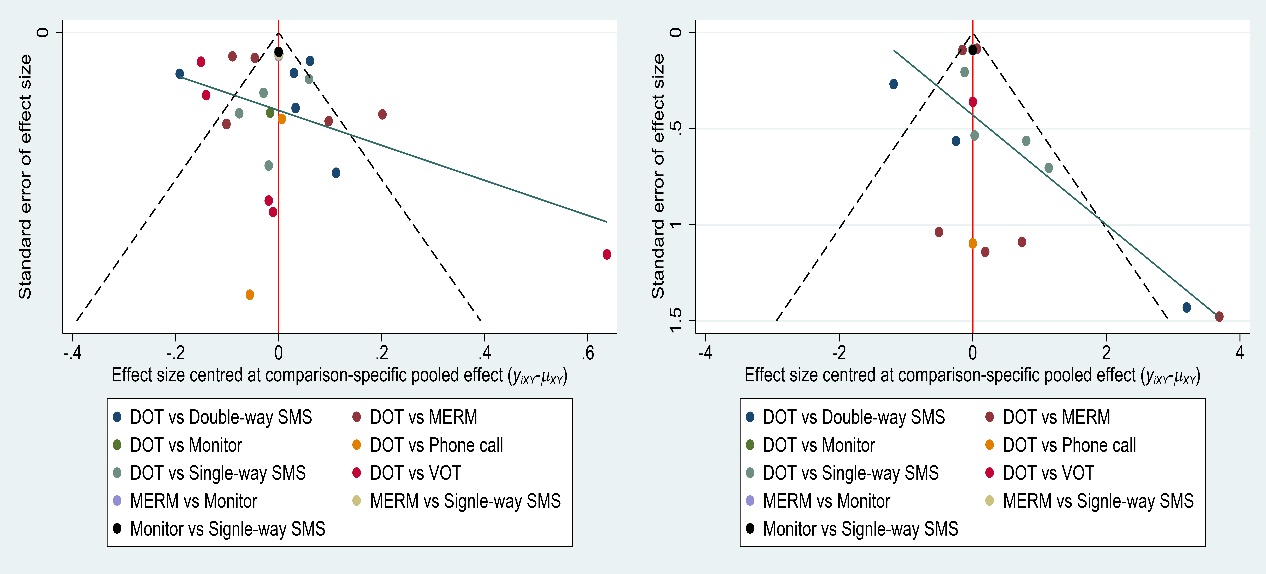
**

# eTable3. Grade of result for conventional meta-analysis

**Treatment success**

| **Treatment** | **Within-study bias** | **Reporting bias** | **Indirectness** | **Imprecision** | **Heterogeneity** | **Incoherence** | **Confidence rating** |
| --- | --- | --- | --- | --- | --- | --- | --- |
| Monitor and reminder | No concerns | Some concerns | No concerns | Major concerns | No concerns | No concerns | Very Low |
| Monitor | No concerns | Some concerns | No concerns | Major concerns | No concerns | No concerns | Very Low |
| Single-way SMS | No concerns | Some concerns | No concerns | Major concerns | No concerns | No concerns | Very Low |
| Double-way SMS | No concerns | Some concerns | No concerns | Major concerns | No concerns | No concerns | Very Low |
| VOT | No concerns | Some concerns | No concerns | No concerns | Major concerns | No concerns | Very Low |
| Phone call | No concerns | Some concerns | No concerns | Major concerns | No concerns | No concerns | Very Low |

**Adherence**

| **Treatment** | **Within-study bias** | **Reporting bias** | **Indirectness** | **Imprecision** | **Heterogeneity** | **Incoherence** | **Confidence rating** |
| --- | --- | --- | --- | --- | --- | --- | --- |
| Monitor and reminder | No concerns | Some concerns | No concerns | No concerns | Major concerns | No concerns | Very low |
| Monitor | No concerns | Some concerns | No concerns | Major concerns | No concerns | No concerns | Very low |
| Single-way SMS | No concerns | Some concerns | No concerns | Major concerns | No concerns | No concerns | Very low |
| Double-way SMS | No concerns | Some concerns | No concerns | Major concerns | No concerns | No concerns | Very low |
| VOT | No concerns | Some concerns | No concerns | No concerns | No concerns | No concerns | Moderate |
| Phone call | No concerns | Some concerns | No concerns | Major concerns | No concerns | No concerns | Very low |

Los to follow-up

| **Treatment** | **Within-study bias** | **Reporting bias** | **Indirectness** | **Imprecision** | **Heterogeneity** | **Incoherence** | **Confidence rating** |
| --- | --- | --- | --- | --- | --- | --- | --- |
| Monitor and reminder | No concerns | Some concerns | No concerns | Major concerns | No concerns | No concerns | Very low |
| Monitor | No concerns | Some concerns | No concerns | Major concerns | No concerns | No concerns | Very low |
| Single-way SMS | No concerns | Some concerns | No concerns | Major concerns | No concerns | No concerns | Very low |
| Double-way SMS | No concerns | Some concerns | No concerns | Major concerns | No concerns | No concerns | Very low |
| VOT | No concerns | Some concerns | No concerns | Major concerns | No concerns | No concerns | Very low |
| Phone call | No concerns | Some concerns | No concerns | Major concerns | No concerns | No concerns | Very low |
